# Supplementary material for: Indigenous vegetables of family Cucurbitaceae of Azad Kashmir: A key emphasis on their pharmacological potential
Source: PLoS One. 2022 Jun 3;17(6):e0269444. doi: 10.1371/journal.pone.0269444 (PMC9165879; doi:10.1371/journal.pone.0269444)
Supplement: Attachment — Submitted filename: Response to Reviewers.docx [file pone.0269444.s001.docx]

**DEPARTMENT OF CHEMISTRY**

**University of Azad Jammu and Kashmir**

**Muzaffarabad, Pakistan**

**Dr. Kulsoom Akhter**

**Department of Chemistry,**

**The University of Azad Jammu and Kashmir, Muzaffarabad, Pakistan**

Emails: [kalsoom.akhtar@ajku.edu.pk](mailto:Srkhattak@gmail.com) Tel: +92 3348479166

**Friday, March 11, 2022.**

**Responses to Reviewers for** '**Indigenous vegetables of family Cucurbitaceae of Azad Kashmir: A key emphasis to their pharmacological potential'**

**Dear Editor**

Thank you for the opportunity to submit a revised version of the manuscript. As requested, we provide here a rebuttal to the reviewer's comments, as well as a detailed description of how we have met their suggestions for improvement. Please note that we have made almost the proposed changes.

Thanks again for your consideration.

**Response to Reviewer: 1**

We would like to thank the reviewer for his valuable suggestions. The manuscript has been revised (marked as red) accordingly and the detailed response is as follow:

**Reviewer** **#1.**

**Comment 1.** Why selected these plants from the whole family?
**Reply.** These plants/vegetables are most commonly consumed by the local people of Azad Jammu and Kashmir region.

**Comment 2.** Provide the importance and recommendations of the study.

**Reply.**	Importance of the study has been provided at the end of introduction and recommendations in conclusion.
**Comment** 3. Cross check the reference with main body text.

**Reply.** All references have been cross checked with main body text.
**Comment** 4. Typographical and English language corrections**.**

**Reply:** Typographical and English language mistakes present in the whole manuscript were checked and tried our best to remove them.

**Response to Reviewer: 2**

We would like to thank the reviewer for his valuable suggestions. The manuscript has been revised (marked as red) accordingly and the detailed response is as follow:

**Reviewer #2 comments**: The abstract is very concise but abbreviation used more. So, correct it. Introduction is very brief. it does not cover whole topic. Add relevant data in introduction in sequence. Methods are designed in good form. Results description showed lack of justifications. Please add suitable discussion. Conclusion is not properly indicated key findings. All References must be formatted according to given format of the journal. Some spelling mistakes are observed. So, I think, major revision of the paper is required

**Reply:**

- **Abbreviations have been corrected where ever possible.**
- **Some more data has been added in the introduction of the revised manuscript.**
- **Following Justifications for the results along with suitable discussion have been added in the results and discussion section and highlighted as red.**

ʺSimilar trend was reported by Xu et al., (2009) and zhang et al., (2018) where peel extract provided the maximum antioxidant potential than pulp and seeds. Variation in antioxidant potential among different fruit tissues of the same vegetable could be due to the presence of maximum amount of phenolic and flavonoid content in peelʺ.

- **Following references have been added in the list of reference and highlighted as red in revised manuscript.**

Xu, G., Liu, D., Chen, J., Ye, X., & Shi, J. (2009). Composition of major flavanone glycosides and antioxidant capacity of three citrus varieties. *Journal of food biochemistry*, *33*(4), 453-469.

Zhang, H., YANG, Y. F., & ZHOU, Z. Q. (2018). Phenolic and flavonoid contents of mandarin (Citrus reticulata Blanco) fruit tissues and their antioxidant capacity as evaluated by DPPH and ABTS methods. *Journal of Integrative Agriculture*, *17*(1), 256-263.

Zou, Z., Xi, W., Hu, Y., Nie, C., & Zhou, Z. (2016). Antioxidant activity of Citrus fruits. *Food chemistry*, *196*, 885-	896.

- **Key findings of our study have been added in conclusion of revised manuscript.**
- **All references have been formatted according to the given format of the journal**
- **Spelling mistakes have been removed up to the best of our observation.**
- **Response to Reviewer: 3**
- We would like to thank the reviewer for his valuable suggestions. The manuscript has been revised (marked as red) accordingly and the detailed response is as follow:
- **Reviewer #3 comments**:

Indigenous vegetables of family Cucurbitaceae of Azad Kashmir: A key emphasis to their pharmacological potential

I congratulate authors for presenting a nice MS. The MS is well written. However there are some issues to be corrected before publishing.

**Comment1.** Grammatical mistake

**Reply:** We have tried our best to remove the grammatical mistakes in the whole manuscript

**Comment 2.** µgm/ mL

**Reply:** µg mL^-1^ have been changed to µg/ mL in the whole manuscript as per the directions of reviewer

**Comment 3.**Genus and species must have gap

**Reply:** Genus and species have been separated by gap

**Comment 4.** Mistake in ref 15 and 26

**Reply:** References 15 (now 21 in the revised manuscript) and 26 (now 32 in the revised manuscript) have been rechecked, corrected and highlighted as red.

**Comment 5.** Please make reference crosschecking for similar type of work,
What is the difference between this work and previous works (Ref 24, Ref 25, Ref 26, Ref 31)?

**Reply: Difference between our work** **and previous work** **has been explained as follows;**

**i. Difference between our work** **and the work of Khatana et al. [Reference No. 24, now 30 in revised manusript]**

**Khatana et al., reported the TPC and TFC only in peel of C. moschata vegetable from cucurbitacae family where as in our study, we included three vegetables frequently consumed in Azad Jammu and Kashmir region. We investigated various tissues of these vegetables including peel, pulp and seeds and compared them to find which tissue has greater TPC and TFC. Moreover, we used aqueous extracts of all studied vegetables whereas Khatana et al. worked on methanolic extracts in their study.**

**ii. Difference between our work** **and the work of Kondhare et al. [Reference No. 25, now 31]**

**Kondhare et al., evaluated and compared phytochemical and antioxidant activities of C. grandis whole fruit extracted with various solvents whereas in our work, we compared various tissue parts of C. grandis for the evaluation of their antioxidant potential. In addition we also included two more vegetable [T. cucumerina and C. moschata] in our study.**

**iii. Difference between our work** **and the work of Liyanage et al. [Reference No. 26, now 32]**

**Liyanage et al. have compared arial parts including leaves, flowers and fruits of T. cucumerina from cucurbitacae familyand compared these parts for TPC, TFC, antioxidant and antiamylase activities whereas in our study, we investigated different tissues of three cucurbitacae vegetables including *C. grandis, T. cucumerina* and *C. moschata.***

**iv. Difference between our work** **and the work of singh et al. [Reference No. 31, now 39]**

**Singh et al., studied four vegetables of cucurbitacae family including C. maxima, B. hispida, C. lanatus and C. melo where as in our study, we selected different vegetables including *C. grandis, T. cucumerina* and *C. moschata.***

**Comment 6.** What is the reason for positive, negative and no correlation of peel, pulp and seed. Is there any evidence from this research?

**Reply: The positive, negative and no correlation among different tests provide information that the antioxidant assays selected in present investigation are feasible and complementary or not to evaluate the antioxidant activities of *C. grandis, T. cucumerina* and *C. moschata* peel, pulp and seeds. Similarly, correlation of TPC, TFC and antioxidant assays verifies the contribution of these phytochemicals to the antioxidant activities highlighting the importance of these compounds in the antioxidant behaviour. The higher the r value, the higher is the correlation of variables.In present study, we observed the positive correlation between ABTS and TPC**(r= 0.998) of peel extracts whereas a moderate and positive correlation between DPPH radical scavenging activity and TPC was observed with r= 0.686. For peel, no significant correlation between ABTS and TFC was observed as p values were higher than 0.05 significance level. For pulp, ABTS showed negative correlation with TPC(r= -0.92) and TFC(r= -0.915). DPPH values also correlated with TPC while no correlation between DPPH and TFC was observed for peel, pulp and seed extracts (Table 6).

**Comment 7.** What is take home message from this study? Please state this nutshell in conclusion.

**Reply: FOLLOWING lines have been added in conclusion to give the take home message from this study;**

**‟ Moreover, in view of phytochemical screening and antioxidant potential presented by these vegetables of cucurbitacae family, their optimum consumption in diet has been strongly recommended to reduce the risk of oxidative stress and its related diseases”.**

**Comment 8.** What is the global/regional inferences of this study?

**Reply:** This research is of relevance to Pakistan and azad jammu and kashmir because of the very high incidence of oxidative stress and its related complications including diabetes, kidney, eye and nervous tissue damage which are the major health issues in the country, particularly in disadvantaged communities. Recent research have shown antioxidant potential of food constituents. Numerous epidemiological studies have indicated that fruits and vegetables rich in polyphenols are an important factor in preventing diseases. ***C. grandis, T. cucumerina* and *C. moschata*** arethe most common vegetables consumed by the people of Pakistan especially in Azad Jammu and Kashmir region and these could be tremendously important sources of antioxidant agents. It is also very important to highlight the vegetables growing in our region due to their chemical aspects as most of our rural population use them. Most of our population have low income and also live without proper medical facilities and rely on the folk medicines for the treatment of different diseases. So screening of our food constituents used in folk medicinal system is of great significance with respect to our regional needs.
