# Supplementary material for: Indigenous vegetables of family Cucurbitaceae of Azad Kashmir: A key emphasis on their pharmacological potential
Source: PLoS One. 2022 Jun 3;17(6):e0269444. doi: 10.1371/journal.pone.0269444 (PMC9165879; doi:10.1371/journal.pone.0269444)
Supplement: Attachment — Submitted filename: Response to Reviewers.docx [file pone.0269444.s002.docx]

**DEPARTMENT OF CHEMISTRY**

**The University of Azad Jammu and Kashmir**

**Muzaffarabad, Pakistan**

**Dr. Kulsoom Akhter**

**Department of Chemistry,**

**The University of Azad Jammu and Kashmir, Muzaffarabad, Pakistan**

Emails: [kalsoom.akhtar@ajku.edu.pk](mailto:Srkhattak@gmail.com) Tel: +92 3348479166

**Monday, April 25, 2022.**

**Responses to Comments for** '**Indigenous vegetables of family Cucurbitaceae of Azad Kashmir: A key emphasis on their pharmacological potential**

**Dear Editor**

Thank you for the opportunity to submit a revised version of the manuscript. As requested, we provide here a rebuttal to the comments, as well as a detailed description of how we have met the suggestions for improvement. Please note that we have made almost the proposed changes.

Thanks again for your consideration.

**Journal Requirements:**

Please review your reference list to ensure that it is complete and correct. If you have cited papers that have been retracted, please include the rationale for doing so in the manuscript text, or remove these references and replace them with relevant current references. Any changes to the reference list should be mentioned in the rebuttal letter that accompanies your revised manuscript. If you need to cite a retracted article, indicate the article’s retracted status in the References list and also include a citation and full reference for the retraction notice.

**Reply.** We have reviewed our reference list and we ensure that it is complete and correct. We also verify that no retracted papers have been cited in the manuscript. Moreover, no changes have been made to the reference list.

**Additional Editor Comments**

The paper albeit has been updated as per reviewers' comments but it needs further improvements. Please correct as:
**Comment 1.** Table 1, see percentage yield is not correct, it should be revised.

**Reply.** The values of percentage yield indicate the percentage of aqueous extracts from the peel, pulp, and seeds of studied *Cucurbitaceae* vegetables. The obtained values are written in Table 1, as well as in the text, section 3.1, and found correct.

**Comment 2.** Table 2, write the full name of the plant in Latin

**Reply.** The full names of plants have been written in Table 2 of the revised manuscript.
**Comment 3.** Table 3, write the full name of the plant in Latin

**Reply.** Changes have been done as per suggestions.
**Comment 4.** revise the English language, get expertise from a Native English speaker or from any Professor of English Deptt of your University and also add his letter.

**Reply.** We tried our best to fix the problem of English grammar.

**Reviewers’ Comments**
**Reply.** No comments from reviewers are found related to the revised manuscript.
